# Global Motions of the Nuclear Pore Complex: Insights from Elastic Network Models

**DOI:** 10.1371/journal.pcbi.1000496

**Published:** 2009-09-04

**Authors:** Timothy R. Lezon, Andrej Sali, Ivet Bahar

**Affiliations:** 1 Department of Computational Biology, School of Medicine, University of Pittsburgh, Pittsburgh, Pennsylvania, United States of America; 2 Department of Bioengineering and Therapeutic Sciences, Department of Pharmaceutical Chemistry, University of California at San Francisco, San Francisco, California, United States of America; 3 California Institute for Quantitative Biomedical Research, University of California at San Francisco, San Francisco, California, United States of America; Stanford University, United States of America

## Abstract

The nuclear pore complex (NPC) is the gate to the nucleus. Recent determination of the configuration of proteins in the yeast NPC at ∼5 nm resolution permits us to study the NPC global dynamics using coarse-grained structural models. We investigate these large-scale motions by using an extended elastic network model (ENM) formalism applied to several coarse-grained representations of the NPC. Two types of collective motions (global modes) are predicted by the ENMs to be intrinsically favored by the NPC architecture: global bending and extension/contraction from circular to elliptical shapes. These motions are shown to be robust against tested variations in the representation of the NPC, and are largely captured by a simple model of a toroid with axially varying mass density. We demonstrate that spoke multiplicity significantly affects the accessible number of symmetric low-energy modes of motion; the NPC-like toroidal structures composed of 8 spokes have access to highly cooperative symmetric motions that are inaccessible to toroids composed of 7 or 9 spokes. The analysis reveals modes of motion that may facilitate macromolecular transport through the NPC, consistent with previous experimental observations.

## Author Summary

The nuclear pore complex (NPC) serves as the sole gateway to the cell nucleus, and its proper functioning is therefore crucial for gene expression and many vital signaling pathways. Although it is typically circular, the overall structure of the NPC has been observed to change in response to the presence of cargo. Recently, the molecular architecture of the yeast NPC, including the shapes and relative positions of its constituent proteins, has been resolved. These new structural data provide us with a first opportunity to construct an accurate dynamical model of a macromolecular machine containing hundreds of proteins. By modeling the NPC as a network of masses connected by springs, we investigate its probable large-scale dynamics. We start from a very coarse model and gradually refine it, observing how the structural details influence the calculated dynamics. We find that the NPC dynamics are quite similar to those of a flexible toroid with an uneven mass distribution, and that the 8-fold symmetry that is universally observed in NPCs enables them to undergo certain collective motions that are inaccessible to structures of other symmetries.

## Introduction

**Figure 1:**
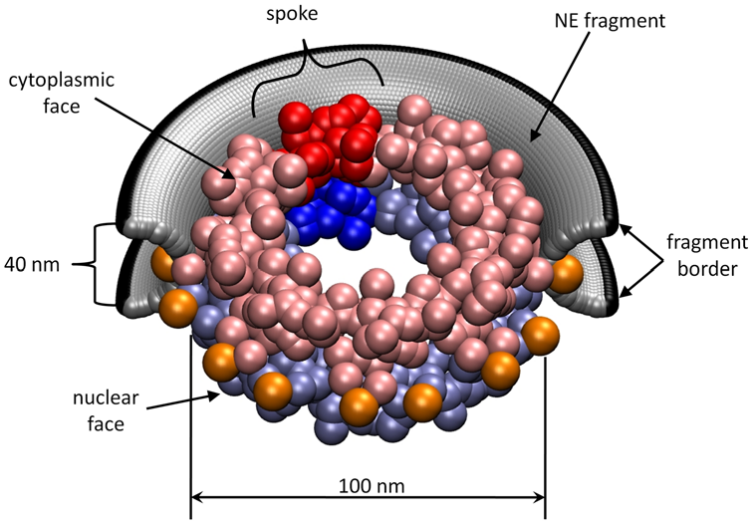
Schematic view of model adopted for the NPC/NE system. The yeast NPC consists of 456 proteins (nucleoporins), shown here as spheres, that form eight identical spokes. The cytoplasmic face of the NPC (facing the viewer) is colored pink, and the nuclear face is colored light blue. A single spoke is highlighted in red/blue. The lumenal ring, consisting of sixteen POM152 nucleoporins is colored orange. Half of the NE fragment is shown in silver, and the border of the NE fragment is colored black.

Any macromolecule entering or exiting the nucleus must pass through a nuclear pore complex (NPC). NPCs are formed by hundreds of proteins organized into cylindrically symmetric pores that act as gateways to the cell nucleus. The NPC has a mass of ∼50 MDa in yeast and up to 125 MDa in vertebrates, comparable to a small organelle. The yeast NPC is a ring of about 100 nm diameter, creating a central pore of ∼30 nm diameter. It contains approximately 450 proteins, termed nucleoporins, arranged into eight similar “spokes” extending from the NPC's central channel to its outer perimeter (Figure 1). Each spoke of the yeast NPC exhibits quasi-twofold symmetry about the lumenal plane of the nuclear envelope (NE), giving the NPC quasi-sixteenfold symmetry and reducing the number of unique proteins in the complex to only about 30. The NPC central channel is coated by several “FG nucleoporins” that characteristically contain multiple structurally disordered phenylalanine (F) and glycine (G) repeats. The FG nucleoporins collectively serve as a selective barrier between the nucleoplasm and the cytoplasm: Small (<5–30 kDa) particles diffuse freely through the NPC channel, but larger particles require the assistance of karyopherin transport factors to pass through the entropic barrier created by the FG nucleoporins. Transport through the NPC is passive, and the rate of material exchange through the NPC can be accounted for by the concentration gradient of the karyopherins and their cargoes [1].

The NPC is somewhat plastic [2] and has been observed to undergo large-scale conformational fluctuations in the form of elongation or dilation under a variety of conditions. Nuclear export of large mRNA molecules seems to be facilitated by dilation of the NPC [3],[4]. Similarly, some modes of viral infection suggest that the NPC channel widens concurrently with import of viral genetic material [5]: Viral infection often includes the nuclear import of intact capsids, as is the case with papovaviruses [6],[7] and hepatitis B virus [8], or import of the intact viral genome, as occurs with adenoviruses [9] and type-1 herpes simplex virus [5]. The NPC also dilates in response to cation concentration [10] and to certain steroids [11],[12], possibly as a mechanism to regulate transport rates [11]. Recent studies with cryo-electron tomography [13],[14] highlight the structural changes of the NPC during transport, indicating that the central channel alters its profile in response to the presence of cargo.

Although conventional models of nuclear transport focus primarily on the local interactions of FG nucleoporins with karyopherins [15]–[21], a number of mechanisms have been proposed to account for the large-scale motions of the NPC [22],[23]. The limiting factor in determining the nature of the global NPC motions has been the resolution of available data. Cryo-EM and electron tomography images of NPCs reveal global features of the complex, while blurring over its finer details. Alternatively, X-ray crystallography captured the structures of several individual nucleoporins to atomic resolution, but it does not provide the relative positions or orientations of the nucleoporins within the assembled NPC. Recently, an integrative approach has been developed to determine the molecular architectures of macromolecular assemblies from diverse data [24]. Combining spatial restraints that account for the NE excluded volume (from EM), nucleoporin excluded volumes (from the protein sequences and ultracentrifugation), protein positions (from immuno-EM), protein contacts (from affinity purification), and the eight-fold and two-fold symmetries of the NPC (from EM), this approach was used to generate a coarse-grained structural model for the yeast NPC, defining the relative positions of its constituent proteins [25]. Although each individual restraint contains little structural information, the concurrent satisfaction of all restraints from independent experiments drastically reduces the degeneracy of the structural solutions and yields pronounced maxima in the localization probabilities of almost all nucleoporins, thus leading to one predominant NPC architecture [25]. This architecture provides us with enough detail to construct a dynamical model of the NPC at the resolution of a single protein, enabling the exploration of the large-scale dynamics of the NPC.

A dynamical model that has enjoyed considerable success in elucidating the machinery of biomolecular assemblies near physiological conditions is the elastic network model (ENM) [26]. In the simplest ENM, each of the constituent bodies in the system is represented as a point mass, or node, and a network is constructed by joining neighboring nodes through elastic edges that supply linear restoring forces to displacements from equilibrium. ENMs are straightforwardly paired with normal mode analysis (NMA) to provide analytical solutions to motions accessible to biomolecular systems under native state conditions. It has been repeatedly shown that the collectives modes predicted by the ENM correlate well with experimentally observed functional changes in structure triggered by substrate binding or activation [27]. This observation supports the idea that biomolecular systems possess intrinsic dynamics that are uniquely defined by their three-dimensional (3D) structures [27], in accord with experimental observations [28]. Further, ENMs are readily extensible to large systems through a variety of coarse-graining techniques, and they have been applied at various degrees of detail to systems ranging in scale from small proteins to supramolecular assemblies such as the ribosome [29],[30] and viral capsids [31],[32]. The robustness of the global modes predicted by the ENMs and their elegant simplicity and scalability lead us to explore the dynamics of the NPC using ENM-based theory and methods. The known agreement between ENM normal modes and molecular fluctuations can be exploited for structural refinement [33],[34]. Starting from an initial structure, one can iteratively construct an ENM and modify the structure until the structure matches the observed low-resolution density data. Such techniques are commonplace in generating atomic coordinates to fit cryo-EM data [35]–[37], and may similarly be used to refine the structure of the NPC.

Here, we examine the dynamics of the NPC at several levels of coarse-graining with a newly developed version of a classical ENM. We begin by modeling the NPC on the coarsest scale possible, i.e., representing the full complex as a flexible toroid. We then increment the level of detail to include non-uniform mass distributions, spokes, and finally the full NPC architecture. We find that the global modes of the NPC are largely captured by the modes of a simple toroid, suggesting that the global dynamics of the NPC does not depend on the details of the NPC architecture. We note that the eight-fold symmetry permits ease of certain cooperative motions that are inaccessible to structures with other rotational symmetries. Two highly robust modes of collective deformation emerge from the analysis, providing insight into the details of NPC motions that are observed with low-resolution tomography [13],[14].

## Results/Discussion

### 

#### There are three dominant modes of a simple toroid

We began by modeling the NPC as a uniform toroid consisting of about 3800 identical point masses spaced approximately 3 nm apart and enclosed in a torus with characteristic dimensions comparable to those of the NPC (major radius of 30 nm and minor radius of 13 nm). Each of the point masses was connected to its neighbors within 12 nm via elastic springs of uniform spring constant, and the normal modes of motion were calculated as described in Methods. The model is intended to approximate a continuous torus of uniform mass density, and its dynamics are insensitive to the exact number of point masses used, provided that it is sufficiently large (larger than approximately 1000).

**Figure 2:**
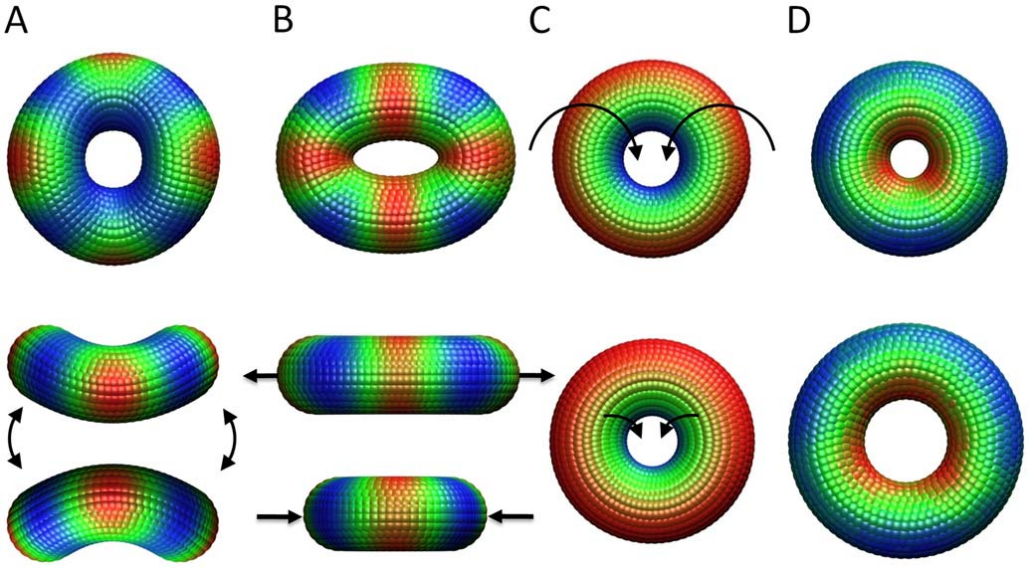
Slow modes of a uniform-density toroid. (A) The first mode is a two-fold degenerate bending of the toroid. The centroids are colored blue to red to indicate increasing mobility. (B) The second mode is a two-fold degenerate stretching of the toroid. (C) The third mode is a non-degenerate rolling mode. The centroids here are colored from blue to red as a function of initial distance from the center of the toroid. The mass centroids on the top (visible) surface move along the toroid surface toward the central channel, and the centroids on the opposite side move away from the central channel toward the toroid perimeter. (D) Mode 6 is a non-degenerate dilation.

A system in thermal equilibrium is expected to fluctuate along all of its normal modes, with arbitrary phase, and amplitudes that are determined by the normal mode frequencies. The modes with lowest frequency are the most easily accessible from energetic point of view, and therefore dominate the global dynamics. The first mode of the homogeneous toroid is a 2-fold degenerate *bending* (Figure 2A). In this mode, the toroid is folded in two directions perpendicular to each other to be distorted into a saddle-like shape. The second mode, which is also 2-fold degenerate, is an in-plane *stretching* of the toroid in one direction and concurrent *contraction* in a perpendicular direction (Figure 2B). This mode has almost no component along the direction of the cylindrical axis. The net effect is to induce a distortion in the shape of the toroid from circular to elliptical. The third is a *rolling* mode that is orthogonal to the toroid's minor axis (circular direction along the center of the ring) (Figure 2C). After the third mode, there is a sharp increase in mode frequency that naturally separates the slowest collective modes from the remaining modes; we therefore focus on the first three *dominant* modes of motion. The remaining slow modes fall into the broad categories of bending, stretching and rolling, but at higher harmonics: The fourth and ninth modes are bending along three and four axes, respectively; the seventh and eleventh modes are stretching along three and four axes. Animations of these motions, as well as those described below, can be found at http://www.ccbb.pitt.edu/People/lezon/research/npc/index.html.

Owing to the symmetry of the toroid, the majority of the slowest modes are two-fold degenerate; these motions are not invariant under rotations about the toroid central axis, and the two-fold degeneracy is required to span the plane perpendicular to the cylindrical axis. Non-degenerate modes in the uniform model, on the other hand, correspond to motions that are invariant under rotations about the central axis. There are three non-degenerate modes among the slowest 20 modes of the uniform toroid model. The first of these is the rolling mode 3 (Figure 2C). The second, mode 6, is a pure dilation (Figure 2D), and the third, mode 18, is a torsional motion leading to counter-rotations of the two toroid faces. It has previously been shown [31] that non-degenerate modes of supramolecular structures play an important role in symmetry-preserving structural transitions. Here, among the two top-ranking non-degenerate modes, the rolling motion (mode 3) may play a role in facilitating the translocation of material through the pore, and the dilation mode 6 may be important for accommodating the passage of large molecules. In mode 3, the toroid surface slips through its central opening, and material attached to the toroid suface would consequently be pulled through the toroid opening under the influence of this mode. Similarly, a dilation like that seen in mode 6 is required to increase the diameter of the central opening if large cargoes are to pass through the toroid.

#### Asymmetric distribution of masses along the axial direction alters the mobilities at the two faces

Next, we investigated the effects of the mass distribution on the toroid modes. We used three models, each of which preserves the particle density and network topology of the uniform-density model, but has a different distribution of mass within the toroid. The mass was made to be a linear function of position such that the minimum and maximum values of centroid masses were the same in all three models.

In the first case, the mass density is a linear function of distance from the toroid surface, such that the toroid's mass is concentrated about its interior and decreases toward the toroid surface. This distribution preserves much of the symmetry of the uniform-density toroid and therefore also reproduces its dynamics. The 10 slowest modes are qualitatively identical to those of the uniform-density model described above, and the mobilities induced by the first three modes have correlations of 1.000, 1.000 and 0.903 with those induced by their counterparts in the uniform-density model. Thus, biasing the mass toward the toroid's interior does not affect the slow modes.

In the second case, the toroid's mass density is made a linear function of radial coordinate, such that the mass is concentrated on the surface facing the central axis. Here, too, the slow modes are qualitatively identical to those of the uniform-density model, and the mobilities induced by the slowest three modes exhibit correlations of 1.000, 0.998 and 0.989 with their uniform-density counterparts. The earliest deviation from the uniform-density model is the order of modes 10 and 12: Although the shapes of these modes are identical in both models, their positions within the eigenvalue spectrum are swapped.

**Figure 3:**
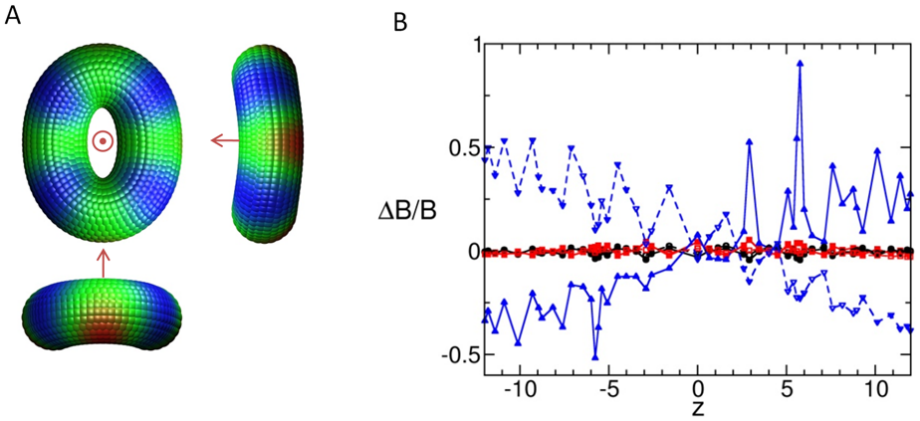
Differences in the mobilities of toroid models induced by mass redistribution. (A) Three views are shown for the second mode of a toroid with axially varying mass density. In each view, the z-axis is shown (by the dot or arrow). The uneven mass distribution causes increased mobility on the lighter side of the toroid (compare to Figure 2B). (B) The fraction change in mobility, ΔB/B, from the uniform-density toroid is plotted against axial coordinate for three toroid models with non-uniform mass distributions: mass distributions linearly dependent on radial coordinate (red squares), distance from minor axis (black circles), and axial coordinate (blue triangles). The solid lines and filled symbols refer to the first mode, which is only slightly altered in the first two cases, as indicated by all points falling close to zero on the ordinate. When the mass is distributed such that the cytoplasmic (z>0) side is more massive than the nuclear (z<0) side, there is an increase in mobility in the cytoplasmic side and a decrease in mobility in the nuclear side relative to the first mode. The opposite effect is observed for the second mode, shown in dashed lines.

A more discernible deviation from the uniform-density model is observed when the toroid mass density varies linearly with the axial coordinate, such that the upper half of the toroid has twice the mass of the lower half. The mobilities induced by the three slowest modes in this case have correlations of 0.931, 0.442 and 0.923 with their uniform-density counterparts. The difference in mobility can be detected starting with the first mode, which preferentially mobilizes the more massive face of the toroid (Figure 3B). A more apparent difference is in the second mode, where the planar “stretching/contraction” of the uniform-density model is accompanied by a preferential mobilization (slight bending) in the less massive face (Figure 3A). This effect is not unique to the second mode; the shapes of all of the degenerate planar modes are altered by biasing the mass toward one face of the toroid.

#### The multiplicity of spokes affects symmetric motions

**Figure 4:**
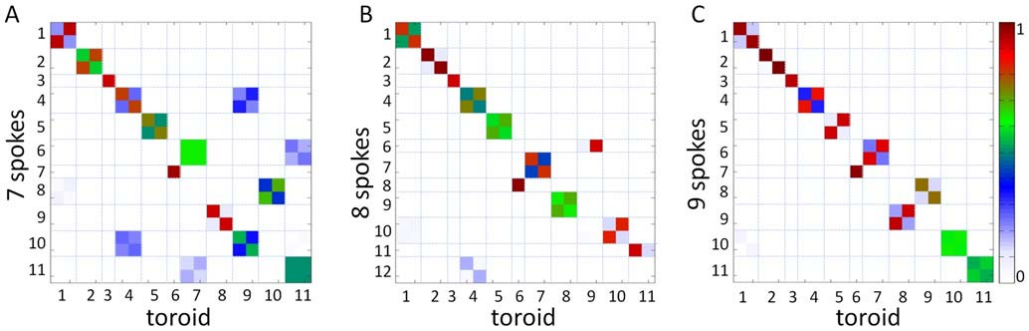
Overlap of global modes for spoke-based models with those of uniform toroid. The three panels show the correlation cosines between the slowest modes of toroids composed of 7 (panel A), 8 (panel B) and 9 (panel C) uniform-density spokes with those of a uniform toroid. Perfect agreement between the modes of two models would be indicated by the unit matrix. For clarity, the blocks corresponding to different modes are separated by thin lines. The 2×2 blocks refer to 2-fold degenerate modes, and the smaller squares within each of these capture the mode overlap as well as an arbitrary phase separation. Here it can be seen that increasing the number of spokes reduces the number of off-diagonal elements in the matrix, indicating a higher overlap between the two sets.

We further considered toroids composed of 7, 8 or 9 loosely connected spokes of uniform mass density. These models were constructed using the same characteristic toroid dimensions as those used for the continuum model, and with an inter-node spacing of 3 nm. To emphasize the physical division of the toroid into smaller components, distinctive gaps of 5 nm were left between adjacent spokes and the inter-spoke spring constant was taken to be 1% of the intra-spoke spring constant. We found that the correlation between the modes of the spoke-based models and those of the uniform-density toroid increases with the number of spokes. Figure 4 shows mode-mode correlations between the three types of spoke-based models and the uniform toroid model. In all three cases, the first five modes are qualitatively identical to those of the uniform toroid.

The differences in the models become more prevalent starting with the sixth mode (Figure 2D), which is non-degenerate in the case of both the uniform toroid and the 8-spoke model. A pronounced difference in the dynamics of the spoke-based models is seen in the population of degenerate slow modes. Eight of the 20 slowest modes of the 8-spoke model are non-degenerate, compared with four for the 7-spoke and 9-spoke models. A non-degenerate mode of the NPC is one that cooperatively induces the same motion in each spoke and is therefore *k*-fold symmetric about the pore's central axis for a toroid of *k* spokes. Such symmetric motions, which include dilations of the entire pore or of some part of the pore, are more easily accessible in the structure of 8 spokes than they are in the structure of 7 or 9 spokes. The prominence of non-degenerate slow modes suggests that the universal 8-fold symmetry of NPCs may have been evolutionarily selected in part due to their ability to undergo concerted deformations with a low energy cost. Symmetry-preserving structural changes play an important role in allosteric regulation [38], and may be of similar value in NPC function.

#### The yeast NPC behaves like a toroid with axially varying mass density

After exploring the intrinsic dynamics of simple toroids and systems of spokes, we investigated the slow modes of the yeast NPC using an ENM. This model highlights how the relative positioning and mass distribution of the nucleoporins impact the inherent dynamics of the NPC. As detailed in Methods, the EN was constructed by using springs of uniform force constant to join all pairs of nucleoporins with mass centers within 14 nm of each other.

**Figure 5:**
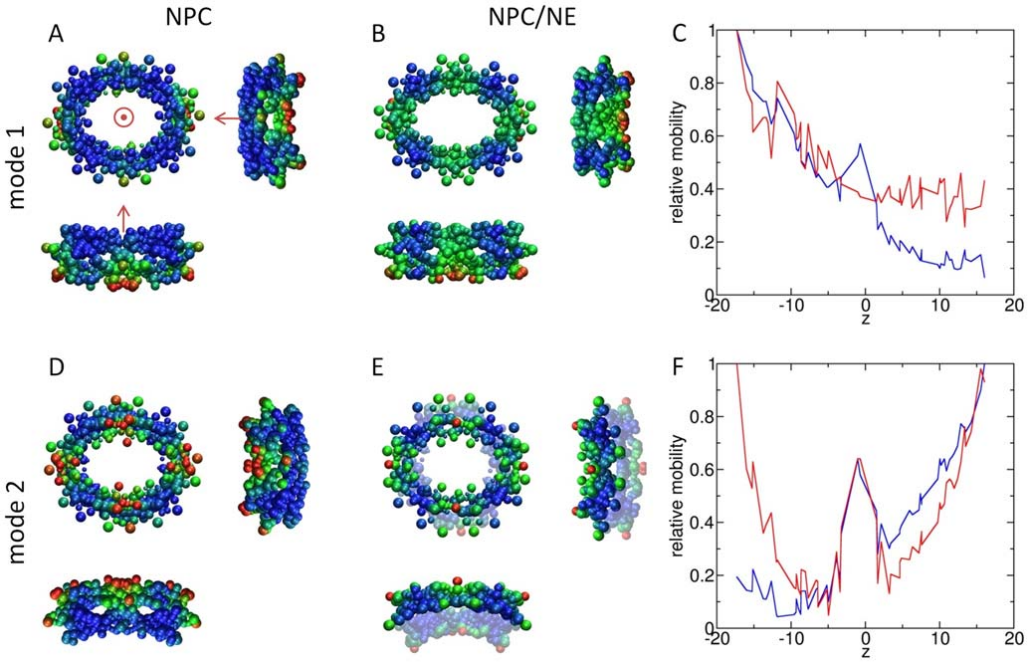
First two modes of yeast NPC. Three views of the two slowest modes of the NPC without (A and D) and with (B and E) the NE fragment are shown. The z-axis is shown in panel A, and orientations are the same in panels B, D and E. Mode 1 is a “stretching/elongation” mode that mobilizes the nuclear face (z<0) more than the cytoplasmic face, whether or not the NE fragment is included in the model. Mode 2 is a “bending” mode that elongates the NPC in the absence of the NE (D), but not when the NE is included (E). For a clearer visualization of the motion, the lower half of the structure is shown in translucid colors in panel E. Scaled mobilities are plotted against axial position in panels C and F for the NPC alone (blue) and the NPC/NE (red). The addition of the NE decreases mobility of the POM rings in the first mode, as shown by the absence of a peak near z = 0 in panel C. In the second mode, the NE creates a more even distribution of motion in the cytoplasmic and nuclear faces, as shown by the peaks near z = +/−12 for the red curve in panel F.

The slow dynamics is dominated by the first two modes (Figure 5). The first is a two-fold degenerate stretching/contraction accompanied by a slight bending, similar to the second mode of the toroid with axially-dependent mass density; and the second is a two-fold degenerate bending. The first mode mobilizes the NPC nuclear face, whereas the second mode mobilizes the cytoplasmic face. The alternating mobilities of the two faces are reminiscent of the alternating access model of transporter proteins that has been proposed as a mechanism of facilitating substrate translocation across the membrane [39]. The shapes of these modes originate at least in part from the NPC's asymmetrical mass distribution; the cytoplasmic face of the NPC, at 16.1MDa, is more massive than its nuclear face, which has a mass of 14.1MDa. Interestingly, these two modes occur in the opposite order as their counterparts in the axially-asymmetric toroid, indicating that the non-uniform shape of the NPC also influences the relative frequencies of the slow modes.

Further comparative analysis reveals that each of the 10 slowest NPC modes can be matched directly with one of the slow modes of a toroid with axially dependent mass density, but this similarity vanishes in the higher frequency modes. In the NPC model, the higher modes tend to preferentially mobilize the least constrained proteins, particularly the 8 copies of Nup85 on the nuclear face and the ring of Pom152 that wraps around the NPC equator (colored orange in Figure 1). The enhanced mobility of these nucleoporins is more likely an artifact of the model. Indeed, one expects that the POM rings are held in place by the NE, and that the Nup85 proteins are in part constrained by the nuclear basket, which is absent from our model. It should be additionally noted that 8 of the 20 slowest modes in this model are non-degenerate, consistent with the results for the model of 8 spokes described above.

#### The NE stabilizes motions about the NPC perimeter

In our final model, we surrounded the yeast NPC with a fragment of the NE. The NE fragment included in our model is only a stand-in for the full NE, which we assumed is placid far away from the NPC. Our NE model consists of 2070 mass centroids, each of which represents an area of about 0.6 nm^2^ on the NE surface. The NE mass centroids are connected to each other and to proximal nucleoporins via elastic springs, and the NE fragment is given periodic boundary conditions as described in Text S1 and shown in Figure S1.

The dynamics are in this case also dominated by the two slowest modes, both of which are two-fold degenerate (Figure 5). Modes 1 and 2 are essentially analogous to modes 2 and 1 of the uniform toroid model (Figure 2), highlighting the significance of the toroidal shape on the selection of intrinsic dynamics. We can also discern the counterparts of the non-degenerate rolling (mode 3) and dilation (mode 6) of the toroid, as the respective non-degenerate modes 4 and 11, although in each case the motion undergone by the NPC is more complex due to its particular distribution of individual proteins within the spokes.

One of the primary effects of the NE on the NPC dynamics is the stabilization of the POM rings. Each of the first two modes of the NPC/NE has 0.87 overlap with its counterpart in the NPC modeled alone, and much of the difference is owed to decreased motion of the POM rings in the presence of the NE. Although the POM nucleoporins are mobile in the first mode of the NPC/NE, in the second mode they move axially but not radially. The reduced motion of the POM nucleoporins is exhibited throughout the slow modes and results in dynamics for the NPC/NE that is not present in the NPC-only model. The tradeoff that comes with increased stability of the POM rings is increased mobility in other outlying nucleoporins, such as Nup82, located on the cytoplasmic face, and nuclear copies of Nup85.

#### Scaffolding nucleoporins are most critical to NPC motions

We next used our model to investigate which nucleoporins have the largest influence on NPC motions. We constructed 30 new models, each lacking all copies of one protein from the NPC, and investigated the resulting dynamics in terms of the dynamics of the unperturbed NPC.

The dominant modes of the 30 structures with deleted nucleoporins have overlap values between 0.534 and 0.920 with their counterparts in the unperturbed NPC. The most significant changes in dynamics coincide with deletions of proteins that are found in the NPC scaffold. The proteins Nup157 and Nup192 each significantly affect the motions and are both centrally located in the NPC and form the “inner rings” of the scaffolding [24]. Nup157 is known to form a complex with Nup170 [33],[34],[40], which is required for normal stoichiometry of FG nucleoporins [41], and likely plays a role in anchoring several FG nucleoporins to the NPC [42],[43]. Nup192 is a scaffold protein that is essential for NPC assembly [44].

Another set of scaffold proteins that affect the NPC dynamics are those that form the Nup84 complex forming the outer rings of the NPC scaffold [45]–[49]. The Nup84 complex is important for NPC assembly, and several of its constituent proteins that are known to influence nuclear transport of large cargoes also affect the modes of the NPC when knocked out. Nup85 is involved in mRNA export and interacts physically with mRNA transport regulators [50],[51]. Nup145, the deletion of which causes the greatest changes in our slowest predicted modes, is known to be involved in both nuclear protein import and RNA export [52]–[56], and also in telomeric transport [57].

#### Equilibrium dynamics suggest alternative conformations of the NPC

The plasticity and large-scale conformational changes of the NPC are topics of considerable recent interest [13],[14],[22]. Cryo-EM studies have suggested that the NPC exists in multiple conformational states [13],[14]. The NPC dynamics are presumably dominated by thermal fluctuations, implying that it moves along its slow modes. Although the details of the slow modes depend on some of the specific parameters of the model, such as the distribution of mass within the NPC, the presence of the FG repeats and the nuclear basket, or the extent of the influence of the NE, the slowest modes for all of the models explored here consist of at least one bending mode and one stretching mode. The stretching mode, and to a lesser extent the bending mode, is consistent with observed elliptical distortions of the NPC [14]. The same authors also suggested that the profile of the NPC central channel varies with the location of the central transporter or cargo [13]. We do not observe any motion that has the sole effect of changing the profile of the central channel, but we propose that the apparent internal restructuring of the spokes may correspond to the bending mode of the NPC. The location of the narrowest region of the central channel will vary with the amount that the NPC is bent, and when averaged over many structures, the bending loses its anisotropy and appears to be a uniform constriction of the pore in one region. If so, then the extent of the bending mode is correlated to the position of cargo in the NPC central channel.

#### NPC dynamics can be refined with higher resolution structures

In the present study, we have examined the global dynamics of the NPC using the recently determined yeast NPC architecture to construct an elastic network model, which in turn has been tuned to retrieve mechanisms of motions that are least sensitive to structural details. Our investigations reveal a set of equilibrium motions that are intrinsically accessible to toroidal structures modeled as elastic materials. As illustrated in Figure 2, the first mode accessible to a uniform density toroid is a two-fold degenerate bending; the second is a two-fold degenerate stretching; and the third is a non-degenerate rolling, each of which may play a role in accommodating or facilitating a cargo transport. Our analysis of NPC motions both with and without the surrounding NE suggests that energetically most favorable motions of the NPC are similar to those of a simple toroid. These motions are modestly altered by the toroid's mass distribution, especially when the mass varies along the axial direction (perpendicular to the membrane), as is the case in the NPC. Another important observation is the dependence of the distribution of motions on the circular symmetry, or the number of spokes that the toroid contains. In particular the 8-fold symmetric toroid is distinguished by a prominence of non-degenerate slow modes, suggesting that the 8-fold symmetry of NPCs may have been evolutionarily selecting for enabling cooperative deformations uniformly distributed among all spokes.

Our study is explorative in that it is based on a theory of normal modes and a low-resolution structural model. Although the structure was determined by satisfying restraints derived from experimental observations about the NPC, its low resolution imposes limits on its accuracy and thus its utility. Nonetheless, many of the gross features of our predictions are reproduced in even coarser models: the mass distribution and 8-fold symmetry of the NPC are the key elements in determining its most likely equilibrium fluctuations. We also had to assume the harmonic potential that we have used in all of our calculations and which provides all of the resultant motions. Similar potentials have time and again proved their worth in coarse-grained dynamical calculations of macromolecules. Our key results are robust to variations in the model and themselves provide testable predictions as to the nature of large-scale motions of the NPC.

To provide a more accurate description of the detailed dynamics of the NPC, our model must be refined using a higher resolution structure, not yet available. Even better descriptions would include information about the elastic properties of the individual proteins, as well as details about the FG repeats in the FG nucleoporins, the nuclear basket, and cytoplasmic filaments. Such refinements inevitably require the accumulation of more data, and efforts are currently under way to produce a higher-resolution structure of NPC. More importantly, our work demonstrates that the dynamics of biological systems can be modeled at the nanometer scale, provided that structural data are available.

## Methods

### 

#### NPC architecture

For the system's equilibrium conformation, we begin with the configuration of 456 nucleoporins in the yeast NPC [24],[25]. The structure is based on an ensemble of 1000 models of the NPC that satisfy restraints taken from experimental data. Each protein in our model is represented by a single point mass, or mass centroid, located at its average position in the ensemble. Any residual deviations from 8-fold symmetry are removed by aligning the eight spokes via rotation about the NPC central axis, repositioning each protein centroid within the spokes at the mass centers calculated over the eight spokes, and reconstructing the full NPC via a second rotation about the central axis. The final NPC structure consists of 456 nodes/centroids that comprise eight identical spokes of 57 centroids each.

#### NE architecture

Because the NPC functions embedded in the NE, we also consider the local effects of the NE when modeling NPC dynamics. The NE consists of two parallel lipid bilayers separated by a ∼40 nm thick perinuclear cisterna. In the vicinity of the NPC, the cytoplasmic and nuclear faces of the NE merge, forming a curved surface that resembles the axial face of a torus with characteristic radii of 54 nm and 15 nm (see Figure 1). To represent the fragment of the NE near the NPC, we used a discrete model composed of partially overlapping spheres of radius 2.25 nm placed on an approximately square lattice with nearest-neighbor separation of about 3.6 nm. Each NE volume element (NE centroid) was given a mass of 31.7 kDa, which gives the NE a mass density of ∼0.9 g/cm^3^. For further details, see Text S1.

#### Elastic Network Model

We propose a variation of the anisotropic network model (ANM) [58],[59] to study the NPC equilibrium dynamics. The system is represented as a set of point masses that interact through Hookean couplings, and the total potential is a sum of pairwise interactions,
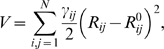
(1)where γ_ij_ is the coupling constant between nodes *i* and *j*, *R_ij_* is the instantaneous distance between these nodes, and *R_ij_^0^* is their equilibrium distance. If **q** is the 3*N*-dimensional vector of Cartesian displacements of the *N* nodes in the system from their equilibrium positions, then the set of *3N* equations of motion reads in compact notation
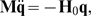
(2)where **M** is the diagonal matrix of masses of the nodes and **H** *_0_* is the Hessian matrix of second derivatives of the potential with respect to the components of **q**. Defining the mass-weighted coordinates 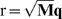, we can rewrite Eq. 2 as
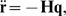
(3)where 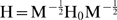 is the mass-weighted Hessian. The nonzero eigenvectors, **v** ^(*k*)^ (1≤*k*≤*3N−6*), of **H** define the directions of collective fluctuations/oscillations near the system's equilibrium conformation, and the corresponding eigenvalues *λ_k_* are the squares of the oscillatory frequencies in units that are determined by the spring constants γ_ij_. The *k^th^* eigenvalue represents the curvature of the energy well along the *k^th^* mode direction [59]. The lowest frequency oscillations thus require the least energy for a given deformation. As a result, motions along those directions are most easily accessible to the system, and usually correspond to the most collective motions. We are primarily interested in analysis of these intrinsically accessible slowest modes of vibration as potential mechanisms enabling/facilitating the translocation of large molecules across the NPC.

We define the mobility, *B_i_^k^*, induced on node *i* by mode *k* as
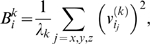
(4)where the component 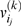
of the eigenvector **v** ^(*k*)^ describes the motion of *i* ^th^ node in the *j* direction.

#### Construction of ENM for NPC

The network topology defined by connecting pairs of amino acids that are separated by a distance of roughly 15 Å or less has been shown in various applications to lead to structural dynamics that agree well with experimental data [60]. No such rules yet exist for constructing networks of supramolecular systems on the scale of the NPC, so we determine them empirically. We determine the network topology by assigning contacts to nodes within cutoff distances based on radial distribution functions. Analysis of radial distribution functions of the nucleoporins and NE centroids suggests that the second coordination shell is at a distance of 10–15 nm for nucleoporins and 7–10 nm for NE centroids.

Using the nucleoporins only, we vary the cutoff distance from 10 nm to 18 nm and calculate the resulting dynamics. We find that the slow dynamics are most robust against changes in the spring constant between 13 nm and 15 nm, and we select a cutoff distance of 14 nm for our model. We separately model the NPC with a fragment of the NE, varying the cutoff distance of the NPC and NE from 12 nm to 18 nm and from 8 nm to 12 nm, respectively. We find that the slow NPC dynamics are least sensitive to the details of the NE boundary conditions when the NE cutoff value is 8 nm, and we empirically select the values of 14 nm for nucleoporin centroids and 8 nm for NE centroids in the mixed NPC/NE models. If the cutoff distance for NE centroids is small compared to that for the nucleoporins, the NE has little perturbative effect on the NPC slow modes, but if the NE centroid cutoff distance is too large, the NPC slow modes are determined almost exclusively by the NE dynamics. Our selected cutoff values permit the NE to influence the NPC slow modes without dominating them. The nodes in the network resulting from our selected cutoff values are each connected to between 13 and 51 neighbors, with an average coordination number of 20. This method of assigning edges sufficiently constrains even the most remote nodes without over-constraining those in the dense regions of the structure. It is also robust against small variations in the selected cutoff distances: Both cutoff distances can be altered by a few nm without significantly affecting the shapes of the slowest modes.

Once the network topology is defined, the only free parameters in the model are the spring constants γ_ij_. In accord with conventional ENMs, we assume that γ_ij_ depends exclusively on the types of the interacting molecules, proteins or lipids, that the particular spring (or network edge) associates. The adoption of different spring constants for different molecules is in accord with previous applications of ENMs to complexes formed by proteins and DNA/RNA, where different force constants have been adopted for amino acid pairs, nucleotide pairs, and interfacial amino acid-nucleotide pairs [60]. In the present case, the edges between two proteins are given spring constant γ_NUP_, those between two volume elements of the NE have spring constant γ_NE_, and those between a protein and an NE node are calculated as 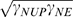. In our model we use the ratio γ_NE_ = 10γ_NUP_, selected to endow the NE with higher rigidity than the NPC.

#### Integrating the effects of the NE

The purpose of including a fragment of the surrounding NE in our model is to further account for the effects of environment on the NPC dynamics; the dynamics of the NE fragment itself are not of particular interest in this study. We therefore employ the following method, detailed elsewhere [61],[62], for constructing a Hessian for the NPC only that still includes the effects of the surrounding NE fragment. The structure of the NPC composed of *N* proteins is described by the 3*N*-component vector 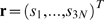, and the structure of the NE fragment composed of *n* mass centroids is given by the vector 
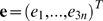
. The full system of the NPC and NE fragment is described by the 3(*N+n*)-component vector 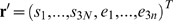  =  (**r** *^T^* **e** *^T^*)*^T^*, where the first *3N* components refer to the NPC, and the last 3*n* to the NE fragment. The Hessian for the NPC and NE fragment together can be decomposed as follows:
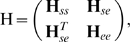
(5)where **H** *_ss_* contains contributions from interactions of the NPC with itself, **H** *_ee_* accounts for interactions of the NE fragment with itself, and **H** *_se_* contains interactions between the NPC and the NE fragment. Minimizing the potential energy with respect to all components of e, one obtains the 3N×3N pseudohessian,
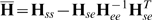
(6)which implicitly includes the effects of the NE fragment. The pseudo-Hessian thus constructed has six normal modes with zero eigenvalue, and 3*N*-6 normal modes corresponding to internal motions of the NPC.

## Supporting Information

Figure S1 Three models of the NE fragment. Top row: Cartoons of the NE fragment models, colored to indicate mobilities induced by the slowest degenerate mode (A) Free boundaries shown at far left, lead to large motions at the border of the NE fragment, indicated by red coloring. (B) Bath-coupled boundaries, center, couple the centroids on the NE fragment border (Figure 1) to two points on the NE axis. (C) Toroidal boundary conditions use a full torus to model the NE fragment. Here, the outside of the torus is transparent for clarity. Bottom row: Average mobilities imposed by the slowest 20 modes on the interior (solid blue) and border (broken red) nodes of the NE fragment. Mobilities are rescaled such that the largest average mobility of an interior node is 1. We seek an NE model that imposes less mobility on the border nodes than on the interior nodes for the lowest modes, and model (C) satisfies this criterion.
(6.15 MB TIF)

Text S1 This file details the models of the NE that were used in this work, as well as the method of comparing motions for structures of different sizes.
(0.11 MB DOC)
