# Supplementary figures and images for: Global Motions of the Nuclear Pore Complex: Insights from Elastic Network Models
Source: PLoS Comput Biol. 2009 Sep 4;5(9):e1000496. doi: 10.1371/journal.pcbi.1000496 (PMC2725293; doi:10.1371/journal.pcbi.1000496)

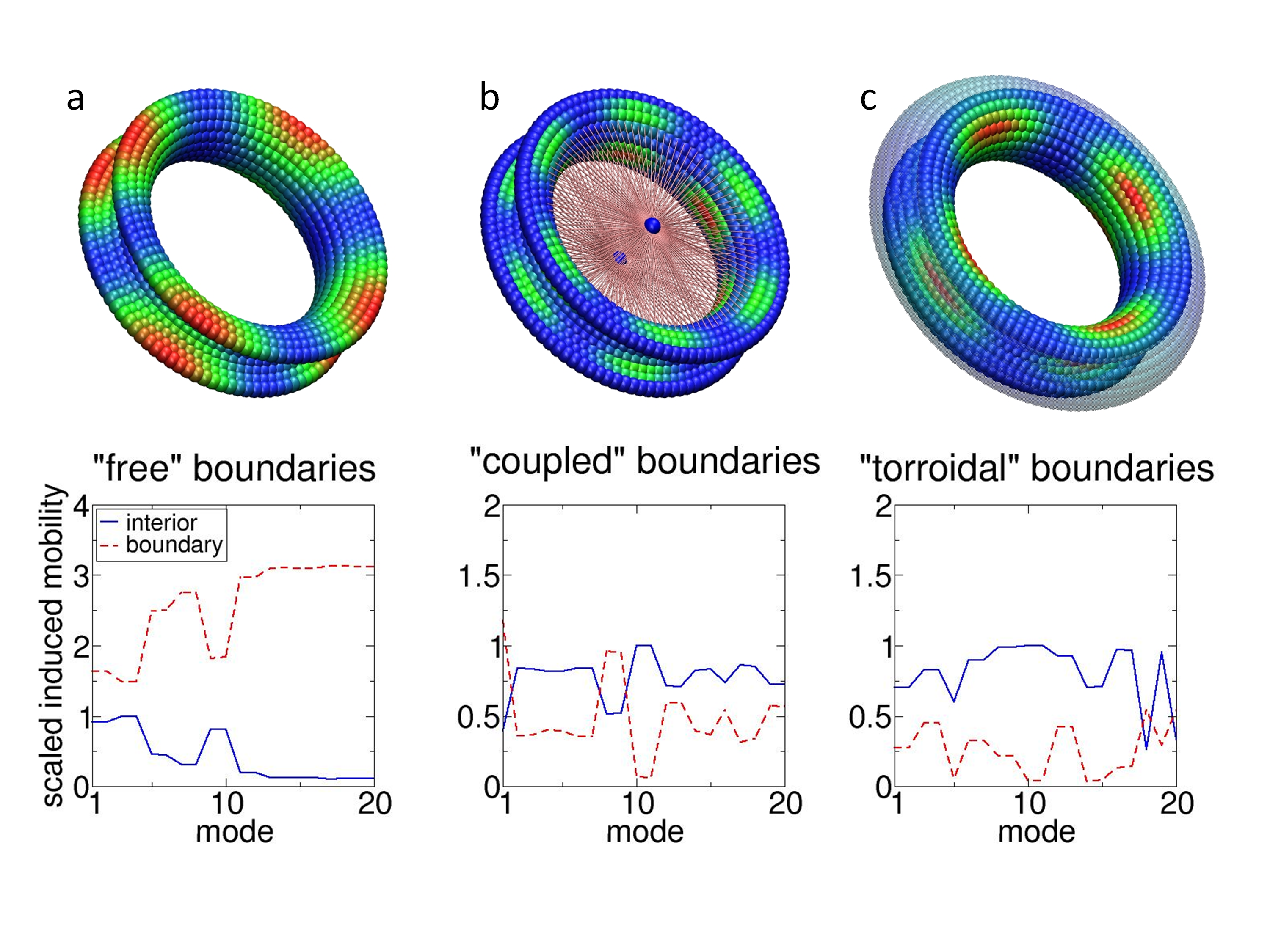

Supplement: Figure S1 — Three models of the NE fragment. Top row: Cartoons of the NE fragment models, colored to indicate mobilities induced by the slowest degenerate mode (A) Free boundaries shown at far left, lead to large motions at the border of the NE fragment, indicated by red coloring. (B) Bath-coupled boundaries, center, couple the centroids on the NE fragment border (Figure 1) to two points on the NE axis. (C) Toroidal boundary conditions use a full torus to model the NE fragment. Here, the outside of the torus is transparent for clarity. Bottom row: Average mobilities imposed by the slowest 20 modes on the interior (solid blue) and border (broken red) nodes of the NE fragment. Mobilities are rescaled such that the largest average mobility of an interior node is 1. We seek an NE model that imposes less mobility on the border nodes than on the interior nodes for the lowest modes, and model (C) satisfies this criterion. (6.15 MB TIF) [file pcbi.1000496.s001.tif]
