# Supplementary material for: Global Motions of the Nuclear Pore Complex: Insights from Elastic Network Models
Source: PLoS Comput Biol. 2009 Sep 4;5(9):e1000496. doi: 10.1371/journal.pcbi.1000496 (PMC2725293; doi:10.1371/journal.pcbi.1000496)
Supplement: Text S1 — This file details the models of the NE that were used in this work, as well as the method of comparing motions for structures of different sizes. (0.11 MB DOC) [file pcbi.1000496.s002.doc]

**Supporting Information**

***NE models.*** The NE is modeled as a set of discrete points connected by springs. The NE mass centroids are spaced 3.6nm apart, such that each mass centroid represents a volume of about (3.6nm)^2^×4.5nm=58.32nm^3^. A typical lipid bilayer is composed of dipalmitoylphosphatidylcholine (C_40_H_80_NO_8_P), which has a mass of 734 g/mol and occupies an area of about 0.6nm^2^/molecule on the membrane surface. We expect to find about 43.2 molecules per NE mass centroid in our model, giving each centroid a mass of 31.7kDa and a density of 0.9g/cm^3^, roughly equivalent to that of vegetable oil.

We consider three models of the NE fragment that respectively use free, fixed and periodic boundary conditions. Under free boundary conditions, the NE fragment border is unrestrained and the NE fragment is modeled with 1070 mass centroids comprising the axial face of a hollow torus (Figure S1A). The ENM is constructed by connecting any two centroids within a cutoff distance of 8nm via a spring of constant γ_NE_. These conditions lead to regions of undesired high mobility at the NE fragment boundary, and similar results are obtained even when the boundary centroids are given masses several orders of magnitude greater than the masses of the interior centroids. We reject this model as excessively flexible.

For fixed boundary conditions, each mass centroid at the boundary of the NE fragment is coupled to a “bath” that is fixed in space. The bath consists of two anchor points on the torus axis, one each in the planes containing the outermost rings of the NE fragments (Figure S1B). Each centroid on the NE fragment boundary is connected to both anchors with a spring of constant γ_bath_. The motion at the NE fragment edges can be controlled by varying γ_bath_: a value of γ_bath_=0 is equivalent to the free boundary conditions described above, whereas γ_bath_ >1000γ_NE_ effectively immobilizes the NE fragment boundary, giving it excessive rigidity. A value of γ_bath_ ≈ γ_NE_ endows minimal flexibility to the NE fragment edges, but the coupling between the nuclear and cytoplasmic boundaries still results in qualitatively unrealistic motion.

The periodic model represents the NE fragment as a hollow torus (Figure S1C) of 2070 mass centroids, each having equal mass. Unlike fixed boundary conditions, which require the adjustable parameter γ_bath_, the periodic boundary conditions need no additional user-defined parameters or tuning. This boundary condition is referred to as periodic because the cytoplasmic and nuclear faces of the NE fragment are coupled via the outside of the torus. The resulting modes of motion exhibit greatest mobility on the axial face of the torus and transition smoothly to regions of little movement near the highest and lowest points on the torus (Figure S1).

We adopt the periodic model for the NE fragment because its low frequency modes are qualitatively realistic and it minimizes the number of arbitrarily defined parameters in the NE fragment model. The cost of the model is an additional 1000 mass centroids, which increase the demand on computational memory; however, the NMA of an elastic network with ~2500 nodes is easily handled with the current generation of processors.

***Comparing modes calculated for different models.*** We are interested in comparing the global motions predicted by different NPC models (below) so as to examine the robustness of the predicted modes. The normal modes form an orthonormal basis on the mass-weighted coordinates of a system, and the inner product of mass-weighted modes can therefore be used to assess the overlap between two sets of modes generated for two different structural models. If $\vec{u}^{i}=\left(u_{1}^{i},...,u_{3N}^{i}\right)$ and $\vec{u}^{j}=\left(u_{1}^{j},...,u_{3N}^{j}\right)$ are two modes belonging to the respective sets, then $C^{ij}=\left|\sum_{k} u_{k}^{i}u_{k}^{j}\right|$ is their overlap. Note that $C^{ij}\in[0,1]$.

Modes of non-identical structural models cannot be directly compared because they are not defined over a common space. To compare two modes of different dimension, then, we must first approximate one in the space of the other. The instantaneous conformation of a structure of *n* nodes is specified by the set of mass-weighted coordinates organized in a 3*n*-dimensional vector, , where $\vec{x}_{i}$ is a 3-component vector. Similarly, mode *j* of the structure is described by a set of 3-dimensional vectors, , representative each of the relative motion induced by mode k on a given node. Because the slow modes are collective and vary little over short distances, we can approximate the value of the mode *j* at any point $\vec{q}$ as the average of $\vec{v}^{j}$in the local neighborhood of $\vec{q}$, or

, (S1)

where the summation is over the *m_q_* nodes in the neighborhood of $\vec{q}$. If we define a second model, , of N nodes, we can find the normalized components of the projection of $V^{j}$ in the space of *Y* as

, (S2)

where the summation is over the *m_i_* nodes of *X* in the neighborhood of $\vec{y}_{i}$, and the factor $\sqrt{n/N}$ ensures proper normalization. The mode $\vec{v}'^{j}$can be directly compared to other modes of the structure *Y*. In this work we consider the neighborhood of a node for projection purposes to be a sphere of 5nm radius.
